# Akatama

**Published:** 1903-09-19

**Authors:** 


### AKATAMA.

DR. F. C. WELLMAN gives an account in the *Journal of Tropical Medicine* of an affection which he has had an opportunity of studying among the natives of West Central Africa. The disease is called by the natives akatama, and as a scientific name for it Dr. Wellman suggests the title of *neuritis peripheralis endemica*. The people affected are the Bantus, who live on a plateau about 5,000 to 6,000 feet above the sea-level, and who are therefore at times exposed to fairly cold weather. About 3 or 5 per cent. of the population are affected. Akatama is found oftener in men than in women, in the young and middle-aged than in the old, and not at all in children and Europeans. The symptoms are: (1) shooting, prickling, "crawling" pains accompanied by numbness in the affected parts, which are usually the legs and arms only. The pain is relieved by the heat of the sun or a fire; (2) erythema, with some swelling which generally subsides as the patient gets warm over a fire or in the sun; (3) a peculiar walk due to a tendency to curl the toes so as to walk upon the heels and ends of the toes. In severe cases the patient cannot walk at all in cold weather. The diagnosis is easy. It differs from beriberi in that there is no pain in the calves, lumbar region, etc., on pressure, and no heart symptoms and no muscular paralysis and atrophy. The peculiar walk is due to pain and is easily distinguished from the gait of ataxia or paralysis. The prognosis is good as to life and general health though uncertain as to relief. No treatment that Dr. Wellman tried succeeded in effecting a cure. With regard to the etiology nothing definite was found by examination of the blood and of the affected tissues which might afford a clue. Dr. Wellman suggests three possible explanations: (1) that the condition is induced by exposure to sudden changes of temperature; (2) that it is the result of "nerve starvation," as the natives are a poorly fed people, and (3) that it is an intoxication analogous to beri-beri. This intoxication, he thinks, might be from micro organisms in the food or soil, or might be accounted for by the fact that the maize which is the staple article of diet is always allowed to partially ferment in the process of preparing it for food.

